# When research animals become pets and pets become research animals: care, death, and animal classification

**DOI:** 10.1080/14649365.2022.2073465

**Published:** 2022-05-13

**Authors:** Alexandra Palmer, Tess Skidmore, Alistair Anderson

**Affiliations:** a School of Geography and the Environment, Oxford University Centre for the Environment, University of Oxford, Oxford, UK; b School of Geography and Environmental Science, University of Southampton, Southampton, UK; c School of Sociology and Social Policy, University of Nottingham, Nottingham, UK

**Keywords:** animal research, animal welfare, care, laboratory, veterinary, companion animal, investigación animal, bienestar animal, cuidado, laboratorio, veterinaria, animal de compañía, recherche animale, bien-être animal, care, laboratoire, vétérinaire, animaux de compagnie

## Abstract

This paper explores what happens to care, and decisions about ending and extending life, when research animals become pets and pets become research animals. To do this, we draw on in-depth qualitative research on (i) rehoming of laboratory animals, (ii) veterinary clinical research, and (iii) the role of the Named Veterinary Surgeon (NVS) in UK animal research. We begin by exploring how (in theory and practice) the ethical, affective, and practical elements of care are split in the research laboratory. We then investigate arguments for and against ending and extending animal life via clinical research and rehoming, highlighting how these activities bring norms and dilemmas around animal death in the laboratory and veterinary clinic to the fore. We conclude by demonstrating the value of investigating borders between animal categories for understanding dilemmas around care and death, and for contributing to emerging literatures within geography around animal care, death, and categorisation. Key contributions of our work include highlighting: how care roles can be split; the importance of considering speculative and in-practice elements of care; the context-dependency and multiplicity of practices of killing in the veterinary clinic and laboratory; and the flexibility and changing nature of animal categories.

## RESUMEN

Este artículo explora lo que sucede con el cuidado y las decisiones sobre el final y la extensión de la vida cuando los animales de investigación se convierten en mascotas y las mascotas se convierten en animales de investigación. Para hacer esto, nos basamos en una investigación cualitativa a profundidad sobre (i) el realojamiento de animales de laboratorio, (ii) la investigación clínica veterinaria y (iii) el papel del Cirujano Veterinario Designado (CVD o NVS por sus siglas en inglés) en la investigación con animales en el Reino Unido. Comenzamos explorando cómo (en la teoría y la práctica) los elementos éticos, afectivos y prácticos del cuidado se dividen en el laboratorio de investigación. Luego investigamos los argumentos a favor y en contra de terminar y extender la vida animal a través de la investigación clínica y el realojamiento, destacando cómo estas actividades ponen de manifiesto las normas y los dilemas en torno a la muerte animal en el laboratorio y la clínica veterinaria. Concluimos demostrando el valor de investigar las fronteras entre las categorías de animales para comprender los dilemas sobre el cuidado y la muerte, y para contribuir a las literaturas emergentes dentro de la geografía sobre el cuidado, la muerte y la categorización de los animales. Las contribuciones clave de nuestro trabajo incluyen destacar: cómo se pueden dividir los roles de cuidado; la importancia de considerar elementos especulativos y de práctica de la atención; la dependencia del contexto y la multiplicidad de prácticas de matanza en la clínica veterinaria y el laboratorio; y la flexibilidad y naturaleza cambiante de las categorías de animales.

## RÉSUMÉ

Cet article étudie ce qu’il advient du care et des décisions concernant l’arrêt ou l’extension de la vie, quand les animaux de recherche deviennent animaux de compagnie et que les animaux de compagnie deviennent animaux de recherche. Pour ce faire, nous nous appuyons sur une recherche qualitative approfondie sur (i) l’adoption d’animaux de laboratoire (ii) la recherche clinique vétérinaire, et (iii) le rôle du chirurgien vétérinaire nommé (NVS - *Named Veterinary Surgeon*) dans la recherche animale au Royaume-Uni. Nous commençons en explorant la manière dont, en théorie et en pratique, les éléments éthiques, affectifs et pratiques du care sont divisés dans le laboratoire de recherche. Nous passons ensuite en revue les arguments pour ou contre l’arrêt ou l’extension de la vie animale par le biais de la recherche clinique et l’adoption, en soulignant la façon dont ces activités amènent au premier plan les normes et les dilemmes autour de la mort animal en laboratoire et dans les cliniques vétérinaires. Nous concluons en démontrant la valeur de l’étude des frontières entre les catégories d’animaux pour comprendre les problèmes entourant le care et la mort, et pour contribuer aux recherches naissantes dans la géographie concernant le care, la mort et la catégorisation des animaux. Les contributions majeures de nos travaux comprennent la mise en évidence de: la manière dont les rôles de care peuvent être divisés; l’importance de la prise en compte d’éléments de care spéculatifs et dans la pratique ; le rapport au contexte et la multiplicité des pratiques de mise à mort dans les cliniques et les laboratoires vétérinaires ; et la flexibilité et la nature changeante des catégories d’animaux.

## Introduction^1^

Imagine a dog who has lived her whole life in a research laboratory, who was used for breeding because she carries a gene needed to maintain the colony. After a lengthy socialisation process involving slowly exposing the dog to novel stimuli she is expected to encounter in the home (hoovers, washing machines, sofas), she is now ready to be ‘rehomed’ by moving to a private home as a pet. The laboratory staff have become very attached to this dog (who they call Molly), so they are simultaneously sad to see her go, apprehensive that she receives as much care in her new home as in the lab, and joyful that Molly has the chance to continue living a fulfilling life now that her service to science has ended. Meanwhile, another dog, Rover, has been enrolled by his owner (Richard) in a clinical trial for a new chemotherapy drug. Rover’s health has been deteriorating, and Richard is desperate to see his old friend have the best possible care, and to spend a little more time together. The specialist vet running the research project explains that the treatment may help Rover continue living a fulfilling life. Regardless, Rover will be helping other dogs, and possibly even humans, suffering from similar conditions. Richard signs a consent form, and Rover begins treatment.

These two scenarios illustrate the central purpose of this paper: to explore what happens to care, and decisions about ending and extending life, when research animals become pets and pets become research animals. In doing so, we build on growing literatures within geography on animal care, death, and classification. Care involves attending to the welfare of others, and can be framed as simultaneously ‘a vital affective state, an ethical obligation and a practical labour’ (Puig de la Bellacasa, 2012, p. 197). It is also speculative in that it ‘encourages intervention in what things could be’ (Puig de la Bellacasa, 2017, p. 66), aimed at bringing about a desired future but simultaneously constrained by what the future is expected to hold. Care is increasingly used as a theoretical framework for exploring moral dilemmas around killing non-human animals (hereafter, animals), including in research contexts (Friese, 2019; Giraud & Hollin, 2016; Greenhough & Roe, 2018; Holmberg, 2011; Roe & Greenhough, 2021; Sharp, 2018). Key concerns in this literature, which we explore in this paper, include firstly the differences between care in practice and care as outlined in rules and regulations (Greenhough & Roe, 2018; Sharp, 2018; Singleton, 2015). A second key concern is how care is not necessarily rewarding and comforting – not only for those who fail to be cared for or about, but also potentially those who are cared for, if care involves violence and exploitation (Giraud & Hollin, 2016; Puig de la Bellacasa, 2012; Van Dooren, 2014). Care can also be challenging for those doing the caring (Roe & Greenhough, 2021), raising a third important matter: who is tasked with care, and why? We explore how the multiple elements of care – ethical, affective, and practical – are distributed among different parties in laboratories and veterinary clinics, given that these elements need not go together.

Even killing can be viewed as care, on farms (Law, 2015), in conservation (Palmer, 2020), and for pets (Morris, 2012) and research animals (Holmberg, 2011). As Mazhary (2021) observes, the topic of animal death offers important insights into how humans understand death, and human-animal (and animal-animal) boundaries. Studies of animal death can therefore offer important insights for both animal geographies and geographies of death and dying, particularly around the spatial nature of death and differential grievability amongst humans and animals. Yet Mazhary notes that this topic has been under-explored within geography. We help to address this by exploring how norms and practices around animal death, including who makes decisions, differ for pets and research animals.

Because we are interested in what happens to care and death as animals move between pets and research subjects, our work also contributes to a growing literature on how animals are socially and legally classified. As human-animal studies scholars have pointed out, classifications are shaped by various factors including perceived sentience, affect, and utility (Herzog, 2010; Hovorka, 2019), and tend to be hierarchical, with certain kinds of animals (e.g., pets) typically treated better than others (e.g., pests). Such hierarchies have been described as ‘sociozoologic’ (Arluke & Sanders, 1996) or ‘zoometric’ (Braverman, 2017) scales. As reviewed by Hovorka (2019), geographers have explored not only between-species inequalities arising from classifications – such as conflicts between domestic and wild animals – but also within-species differences. Cows, for example, are treated very differently when viewed as wildlife rather than agricultural animals (Lorimer & Driessen, 2013). This geographical work has highlighted how classifications and within-species inequalities are shaped by spatial location: animals maybe ‘out of’ or ‘in’ their perceived place (Philo & Wilbert, 2000), with location in turn affecting legal status (Braverman, 2013).

However, as Hovorka (2019) points out, efforts to explore inequalities between animals are far less common than discussions of human-animal inequalities. Thus, despite progress towards understanding the causes, consequences, and nuances of sociozoologic/zoometric scales, more work on the subject is needed. In particular, relatively little work has focused on the interface between research animals and pets. Scholars have explored how bonds develop between laboratory workers and animals, with individualised laboratory animals sometimes taking on a pet-like status through being named, spared from euthanasia, or taken home by research staff (Birke & Arluke, 2007; Friese, 2019; Giraud & Hollin, 2016; Greenhough & Roe, 2018; Herzog, 2010; Sharp, 2018). Others have investigated connections between companion and research animals through the development of veterinary medicine and ‘One Health’ approaches (Cassidy, 2016; Gardiner et al., 2015), though there has been little exploration of clinical trials enrolling pets.

Looking at rehoming and veterinary clinical research side by side adds several new insights to these literatures. First, while ethical issues in veterinary clinics and laboratories have been explored, directly comparing these settings brings the norms and practices of care and death applied to pets and research animals into relief. As Sharp (2018, p. 6) observes, boundaries (including, we suggest, between animal categories) are sites where professional and personal moral dilemmas proliferate, and are therefore good places to look when exploring dilemmas around care and killing. Second, these case studies can help reveal the complexity and context-dependency of the creation, maintenance, and welfare implications of animal categories. For example, we show that while animal categories involve some combination of social, legal, ethical, and spatial norms, these elements do not necessarily go together. Furthermore, the animal welfare implications of categories might confound expectations, with research subjects in some instances receiving (arguably) better treatment than pets. As such, our work adds nuance to discussions in geography around animal classifications, hierarchies, and ethics.

We explore care and death at the pet-research animal boundary through two key themes, which arose from our parallel strands of research. We begin by considering which humans are charged with making decisions about care and death in rehoming and veterinary clinical research, and why they are granted this responsibility. Although a detached, ‘neutral’ party is theoretically in charge, in practice care decision-making tends to be complex and context-specific. We then explore debates about whether ‘good care’ involves humane killing or extending animal life: a complex and contested question, with answers dependent on specific contexts, personal values, legal and professional norms, and how people imagine an animal’s future. In concluding, we demonstrate the value of examining boundaries between pets and research animals for better understanding animal care, death, and categories.

## Methods

This paper is based on qualitative research in UK animal research communities, undertaken as part of *The Animal Research Nexus* (AnNex; see www.animalresearchnexus.org): a collaborative, interdisciplinary project seeking to understand historical and contemporary factors that have shaped the UK’s social contract around animal research.

We draw on three strands of AnNex. The first centres on the rehoming of laboratory animals, to private homes, wildlife sanctuaries, zoos, aquariums, and farms. This research was primarily undertaken by TS and involved 28 semi-structured interviews with animal research facility staff (17, 60%), rehoming organisation employees (8, 29%), and individuals who had rehomed animals (10, 36%; some individuals belonged to more than one of these groups, e.g., facility staff who personally rehomed animals). It additionally involved informal conversations and ethnographies at numerous animal research facilities. The second strand, primarily undertaken by AP, focused on non-laboratory animal research, including work at wildlife and fisheries field sites, farms, zoos, and veterinary clinics. It involved interviews with 30 participants, and informal conversations with 24 others; 9 (17%) of these conversations focused primarily on veterinary clinical research, while 10 (19%) were with individuals with experience across research venues. Participants included researchers, regulators, and vets, but no animal owners. The project also involved participant-observation, including during a veterinary clinical trial, and a stakeholder workshop on non-laboratory research (Palmer et al., 2020). The third strand, analysed by AA, focuses on the role of NVSs in animal research laboratories, and how these veterinary roles are constituted, enacted, and challenged, involving in-depth interviews with 33 NVSs. Fieldwork for these projects took place between March 2018 and August 2020, with some interviews taking place remotely over phone, video call, or written correspondence due to participants’ preferences and COVID-19-related restrictions.

Because of the sensitive nature of the topic, we have adopted a policy of anonymisation and de-contextualisation. All interviews were conducted with written consent from participants. Ethical approval was granted by the University of Southampton Ethics Committee (Submission Number: 32026), the Central University Research Ethics Committee of the University of Oxford (Reference Number: SOGE 18A-7), and the University of Nottingham School of Veterinary Medicine and Science (Approval Number 1800 160608).

## Who decides what good care looks like?

We begin by asking: who makes the difficult decisions about care and death in rehoming and veterinary clinical research? On what grounds are they granted this responsibility, and what can this tell us about conceptions of care for research animals versus pets? We begin by examining how ethical, practical, and affective elements of care are in theory split in the laboratory, before exploring discrepancies between care as outlined in law and in practice.

### Splitting care

The UK’s Animals in Scientific Procedures Act (A(SP)A) (1986), overseen by the Home Office, regulates the treatment of research animals. Under A(SP)A, decisions about rehoming and euthanasia are made by the named veterinary surgeon (NVS), whose primary role is to offer ‘independent advice on animal welfare’ within a research institution. Given this role, A(SP)A guidance indicates that it is especially important for NVSs to avoid ‘conflicts of interest that may affect their judgment’ (Home Office, 2014, p. 24). As a professional vet not responsible for the animals’ daily care, the NVS may lack the detailed knowledge of individual animals that people responsible for daily care develop (Greenhough & Roe, 2018). For example, NVS Owen observed that when he visits the laboratory, the Named Animal Care and Welfare Officer (NACWO) will often point to an issue experienced by an animal, such as a lame leg:

they say, “[…] can’t you see it?” and I say “No, because you look at mice every single day and I only look at them once in a while”. So I will always defer to their greater experience and if they say it’s lame, I’ll take their word for it – and usually I can see it once they’ve pointed it out to me. (Interview, 23 May 2018)

However, this detachment means that NVSs are also expected to not be conflicted by the emotional bonds that commonly develop between laboratory animals and their caregivers (Greenhough & Roe, 2018). As NVS Sophie explained, the idea is to ensure that when it comes to decisions about rehoming, ‘you’re doing it for the right reasons […] you have to be assured in your own mind that it’s appropriate for the animal’ (interview, 17 October 2018).

Thus, while the NVS takes lead on the *ethical* element of care, which involves a sense of ‘obligation to look after another’ (Van Dooren, 2014, p. 291), A(SP)A grants them this responsibility on the assumption that the *affective* and *practical* elements of care are performed by someone else. This arrangement echoes long-standing discussions about how best to reconcile ‘emotion’ – usually used with reference to ‘romantic, compassionate and tender sentiments’ rather than less tender emotions like anger – with ‘practical reason’: the process of deciding, through reflection, on the best course of action (Midgley, 1983, p. 1). For example, as Palmer (2020) demonstrates, there is a widespread assumption that attachments to individual animals can impede decision-making about where best to invest scarce conservation resources. To some extent, so too in A(SP)A: NVSs are given authority in part on the assumption that they will not have developed affectionate bonds, even though this means they may lack knowledge of the individual animal. Indeed, the emotional strain arising from the affective elements of caring for both animal-patients and owner-clients in general practice has been cited as a reason for veterinary professionals to move out of general practice and into NVS work (Anderson & Hobson-West, 2022).

Because of this arrangement under A(SP)A, signing one’s pet up for a veterinary clinical trial means signing over decision-making about care and death to the NVS. Whether animal owners realise they are doing this is, however, less clear. Owners are required to sign consent forms before their pet participates in a clinical trial under A(SP)A, and researcher Charles indicated that the Home Office asked him to clearly state in his consent form that ‘in these circumstances we would insist on euthanasia’ (interview, 26 June 2019). However, not all veterinary clinical trials are conducted under A(SP)A – a complex situation that we have explored elsewhere (Palmer et al., 2020, 2021) – so consent forms may receive minimal external scrutiny. Furthermore, there may be other factors that persuade owners to enrol their pets in clinical studies. Veterinarian Charlotte conveyed her impression that specialist offices – where research is typically undertaken, alongside university-affiliated animal hospitals – can be somewhat ‘dazzling’ for clients compared with the more ‘low key’ high-street clinic they would normally visit, and clients may seek to impress specialist researchers who may seem like their only hope for curing a beloved pet (interview, 5 December 2019). Charlotte also observed that clinical trial consent forms can be ‘impenetrable’ and not necessarily accompanied by a clear information sheet. Thus, Charlotte noted that it’s possible owners ‘don’t read it, because it’s like reading the terms and conditions of Amazon […] you trust the person’. This impression is supported by research showing that the majority of the 165 clients surveyed at a UK referral hospital did not read the consent form, since they trusted the vet. Yet the majority also did not feel in control or reassured, and a third reported feeling frightened by the process (Whiting et al., 2017). For these reasons, veterinary professional Elaine argued, based on her personal experience of clinical research (both under A(SP)A and not),

It varies greatly depending on institutions and individual clinicians, but it’s all very poorly handled, and I would argue that none of it is informed consent. That’s a view that isn’t held by every vet – you’ll find vets that don’t agree with that view – but that’s my view. (Interview, 11 January 2019)

Owners may therefore not necessarily understand the full implications of having signed their pet up for a clinical trial, including that, if conducted under A(SP)A, they no longer have the final say on when to euthanise. This is in direct contrast to the ordinary veterinary clinic, where the owner has the final say over care and euthanasia decisions, even if the vet disagrees. Rollin (2011) proposes that while previously disagreements usually arose when clients requested ‘convenience euthanasia’, today conflicts may additionally arise when owners are unwilling to euthanise suffering animals, the idea being that emotional attachments to their pets cloud their judgment about what is best for the animal. Vets may take a range of approaches to resolving such disagreements, including persuasion, confrontation, and acquiescence (Morris, 2012). Potentially shaping such approaches is a philosophical question about the vet’s role. As Rollin (1978) puts it, you could see the vet as like a mechanic, hired to do the client’s bidding, or a paediatric doctor hired to serve a patient who cannot give informed consent. While some acknowledge that owners are most familiar with their pets’ behaviour and therefore in possession of expertise useful for making decisions (Burrow, 2017), others argue that vets should have the final say, since owners may make decisions based on their personal interests rather than what is best for the animal (Gray et al., 2018).

The view that vets should have the final say was sometimes expressed by our research participants as a call to make companion animal treatment more like the laboratory, by separating the ethical element of care from the practical and affective. For example, veterinary professional Elaine argued, ‘when you’re working with a research animal, you make your decision entirely based on what’s in the animal’s best interest. And I would argue that you should be doing it in a clinic, but that’s absolutely not what we do’ (see also Clutton et al., 2017).

While clinical research represents a fulfilment of the wish to have vets responsible for decision-making, the reverse occurs in rehoming, with responsibility shifting from the NVS to the animal’s new owner. This was a source of concern for some laboratory staff involved in rehoming. As NVS Megan observed, ‘With rats and mice, people don’t always take them to the vet as quickly as they should if they’re ill. Whereas, in a lab, if an animal was ill, they would be treated straight away’ (interview, 13 November 2018). It was hoped that new owners would take good care of their rehomed animals. However, once rehomed, animals are treated as ordinary pets in that even if a vet recommends against something (e.g., a life-extending treatment that is likely to cause suffering), it is up to the owner (Morris, 2012). Care practices were therefore perceived as less certain and consistent for pets than research animals. NVS Nadir summarised:

I think a companion animal has a bit more of a lottery in their existence perhaps than these research animals. A cat can be lucky and have good owners, or a cat can be unlucky and have owners that treat them very badly. In this environment [the lab] that doesn’t happen. (Interview, 22 November 2018)

The laboratory was thus viewed as having greater standardisation of care, due to the processes and oversight in place, and division of care labour such that final decisions are in the hands of the NVS rather than the owner. For this reason, rehoming involves speculation and uncertainty on the parts of laboratory staff. In contrast, veterinary clinical research involves vets reclaiming control of care decisions from animal owners – though potentially in a somewhat awkward, poorly communicated manner.

### Practice and theory

Although laboratory care is in theory quite standardised, the practice is more complex. For example, NVSs may become emotionally invested in animals, prompting a need to break away from the typical process outlined in A(SP)A. This is unsurprising, given that bonds nonetheless develop in many contexts where people attempt to remain unattached to animals, such as in farming (Wilkie, 2010), wildlife research (Candea, 2010), and even laboratories where bond-formation has sometimes been viewed as problematic (Giraud & Hollin, 2016). Bonds might also be most common with traditional companion animals (cats, dogs, and horses), which are most commonly considered as rehoming candidates despite making up just a small fraction of animals kept in laboratories: a situation resulting from imaginaries of public concern; the number of homes available for each species; cultural perceptions of companion animals in the home; and aesthetics, with traditionally ‘cute’ animals being better rehoming candidates (Skidmore & Roe, 2020). Such bonds may lead an NVS to rehome an animal personally. For example, NVS Ella explained the conflict of interest that emerged when she elected to rehome a dog:

I remember that slight tension around the fact that you know, it was me saying he had to be homed and then also me saying I had to take him […] [I]t became very difficult to maintain the position that I wasn’t doing this just for my own good to get my dog quicker, but that I was trying to be professional at the same time and make the right decisions. (Interview, 5 November 2018)

When such conflicts arise, an independent party may be brought in. For example, NVS Megan rehomed guinea pigs after she ‘ended up feeling sorry for them. I got one of the other NVSs to do the health check so there wasn’t the conflict of interest.’ This flexible approach served to keep the affective and ethical elements of care separate, thereby upholding a principle of care embedded in A(SP)A.

Care in practice may break away from the theory in other ways as well. For example, decisions about care and death in clinical trials are not necessarily made exclusively (or even primarily) by the ‘neutral’ NVS. In veterinary clinical research under A(SP)A, the ordinarily ‘triadic’ relationship between vets, clients, and animals (Sanders, 1995) becomes a four-way relationship, since the role of vet is split in two: (i) the NVS; and (ii) the researcher, a vet who typically specialises in the condition under study. In theory the former is responsible for euthanasia decisions, but in practice the latter may also be involved. As Home Office inspector Gail summarised, often ‘the NVS would largely be abstracted from that straight decision-making ‘cause they wouldn’t actually have anything to add’ (interview, 15 May 2019). The vet-researcher may therefore be involved in animal care decisions because, while they are technically conflicted – their primary role is to care for the *science* – they also hold considerable expertise on the animal’s condition. Furthermore, this conflict of interest may in practice become minimally apparent if there is a good working relationship between the two vets. While some expressed concerns that the researcher is ‘automatically biased’ (interview with veterinary professional Elaine), others suggested that in practice researchers ‘are pretty professional about it’ and ‘know that they have to have independent input’ (interview with researcher Charles).

Several participants added that in practice it is quite rare for the NVS to insist on euthanasia without the agreement of the animal’s owner. This is in part because, as correctly guessed by vet Charlotte, clinical research typically involves ‘pets with pretty bad diseases where there maybe isn’t another option and this is the last chance that pet has, or maybe that the pet’s fine and […] there’s going to be relatively minimal harm associated with it’. Thus, euthanasia decisions in clinical research might be either highly unlikely, or viewed as an inevitability. It is possible that veterinary clinical research under A(SP)A can only comfortably occur when one of these two scenarios applies and conflict between owners and vets over euthanasia is expected to be minimal. Some participants also reflected that when it comes to clinical research and experimental veterinary treatments, decisions about euthanasia should rely on the classic ‘skills of diplomacy’ central to all veterinary clinical practice (interview with veterinary professional Elaine; see, Morris, 2012 for more on these skills). In short, care in practice in clinical research might mean making owners feel included in euthanasia decisions, thus continuing to treat animals as pets even though their primary legal status is now as research subjects. Animal classifications can therefore be sticky, following animals via social relations into spatial and legal contexts where their classificatory status has changed.

## When does good care involve ending or extending life?

We now turn to the question: under what circumstances is death viewed as good care, for pets, research animals, and those transitioning between these categories? We begin by outlining commonly identified differences between killing animals in the veterinary clinic compared with the laboratory, and where rehoming and veterinary clinical trials sit in relation to these norms. We then examine arguments for and against ending animal life versus extending it via clinical research and rehoming.

### Killing in the clinic and laboratory

Killing in many contexts is framed as a form of care. Preventing suffering during the process of death, or ‘killing well’, is considered important for many involved in killing animals, such as laboratory workers. Killing might also be considered a form of care for some sort of cause or purpose, such as science – hence, common use of the term ‘sacrifice’ to describe the killing of research animals (Birke & Arluke, 2007; Greenhough & Roe, 2018; Holmberg, 2011; Sharp, 2018). Killing might also be regarded as care for the individual animal, if it is believed that the animal would experience considerable suffering in future (Morris, 2012; Yeates, 2010). It is sometimes proposed that in companion animal medicine there is a greater focus on killing when death is in the animal’s best interests, since pets are increasingly treated as family members deserving the best possible care. Meanwhile, in other settings (e.g., the farm, laboratory, or zoo) a more ‘pragmatic’ approach is adopted whereby animals are killed when they are no longer of use. In other words, killing in the veterinary clinic is often undertaken as care for the individual animal ‘patient’, while in the laboratory animals are killed painlessly in service of science rather than their individual interests. However, this distinction is too simplistic, and some vets would rather see the clinic become more like the laboratory in some respects (Clutton et al., 2017; Gardiner et al., 2015; Grimm et al., 2018; Herzog, 2010; Morris, 2012; Rollin, 2011; Sanders, 1995).

Both rehoming and clinical research are often framed as having multiple benefits. For laboratory staff, rehoming offers hope and helps avoid feelings of sadness and guilt embedded in euthanasia, which are common at animal facilities (Bennett & Rohlf, 2005). New owners may gain a sense of personal satisfaction and a sense of ‘saving themselves’ by rehoming ‘rescue’ animals (Weaver, 2013: 698). Meanwhile, veterinary clinical research might benefit: owners desperate for a cure for their pet; laboratory animals, who are not bred or used if pets with naturally occurring diseases are used instead (Palmer et al., 2020); members of the same species, whose health is improved through the use of more accurate disease models (Nature Editorials, 2016); and humans, if you follow the (somewhat vague and contentious) notion of ‘One Health’ (Cassidy, 2016).

Still, in both cases a central purpose is to extend life and therefore benefit the individual animal. Indeed, barriers are in place to prevent rehoming and clinical research from occurring if they are perceived to not be in the animal’s interests. They are therefore instances where, arguably, animal research laboratories adopt a somewhat patient-centric, clinic-like ethic in which the primary concern is the individual animal’s interests.

### Ending life

Extending animal life via rehoming and veterinary clinical research might sound inherently positive. However, some argue that ending life is preferable in certain cases, specifically when animals are expected to suffer (Morris, 2012; Yeates, 2010). A common assumption, which underpins ‘pragmatic’ attitudes towards killing in the laboratory and elsewhere, is that ‘death is not a welfare issue’, i.e. the *process* of death might involve pain and suffering, but the *state* of death is neutral for an animal’s welfare. This idea is embedded in many animal welfare laws, including A(SP)A (Yeates, 2010). Humane killing is common under A(SP)A because it eliminates suffering and is therefore viewed as promoting the 3Rs – encouragement to *reduce* of numbers of animals in research, *refine* procedures to minimise suffering and improve welfare, and *replace* animals with other methods of testing – specifically by *refining* the animal’s experience (Franco & Olsson, 2016). The idea that death is not a welfare issue may also be internalised by those making decisions about care and death in the lab. As NVS Olivette put it:

I try and drill it into people that there’s no poor welfare in death, so if the animal’s looking sick, contact a researcher. “Do you want it?” No, we cull it. Yes, why? How long do you want it for? Then we determine what we’re going to do about it. (Interview, 22 November 2018).

This view of death shapes rehoming guidance and practice. Guidance offered by Directive 2010/63/EU (which applies to animal research across the European Union, and was transposed into A(SP)A) states that animals can be rehomed if: ‘the state of the health of the animal allows it’, ‘there is no danger to public health, animal health, or the environment’, and ‘appropriate measures have been taken to safeguard the well-being of the animal’ (European Commission, 2013). The Directive therefore makes explicit that those animals whose welfare would be compromised if rehomed should be killed at the end of experiments. Thus, in order to rehome, those working in animal research facilities must be sure that the animals will be properly taken care of post-rehoming (LASA, 2002).

But it can be difficult for laboratory staff to ensure that no future suffering will occur during or after rehoming, with animals potentially experiencing suffering during transport from the laboratory, or as a product of the social and/or physical environment in their new home (Prescott, 2006). This may be one reason (alongside others such as personal attachments to animals) why a large proportion of rehomed animals, particularly rodents, are rehomed by laboratory staff and their families, who are trusted to offer appropriate housing and care (Skidmore & Roe, 2020). If present or future imagined suffering cannot be ruled out, legally speaking euthanasia exists as the ‘default’ option. Laboratory care stuff must therefore engage in a process of imagining what care could look like. This involves assessing the logistics of transport to the new home, and the animal’s condition and expected future wellbeing – for example, based on which experiments it was involved in (experiments with long-lasting implications for health will render the animal unrehomeable), and assessments of how well the animal is expected to adjust to the home environment. It also involves assessing whether the potential owner has the ability to offer good care, evidenced by things like income, previous species experience, and available space. Such assessments are complex, and facilities often have only limited resources available to do this, contributing to the relatively small numbers of animals rehomed (Skidmore & Roe, 2020).

Euthanasia is not only the default, legally and practically, but is also sometimes preferred, where there was a perceived risk of the animal suffering. Jane, who works at a rodent facility, explained that with some animals she ‘wouldn’t [rehome] now […] because of welfare reasons’. Jane cited the example of curly-haired mice, which

people used to be like, “oh, they would be nice mice to keep”, but actually they have, not only curly fur, but also curly eyelashes which means they get slightly sore eyes. You wouldn’t want to keep them for anything other than research purposes, otherwise it’s not fair to the animals. (Interview, 30 August 2018)

NVS Olivette similarly reflected on the difficulty of keeping some animals alive for extended periods, such as a particular strain of mice with ‘bad teeth’ that require trimming. Olivette explained that, ‘I’m not happy to cut for a lifetime, you can’t, it’s all the damage you do with the splintering and everything else’; she therefore is only happy to trim teeth up to three times before euthanising the mouse. Certain animals, which are purpose-bred for research, are therefore inherently unsuitable for rehoming or otherwise keeping alive beyond the research period because of their phenotype. Rehoming such creatures could be viewed as a kind of ‘misguided benevolence’ that would lead to unnecessary animal suffering (Koch & Svendsen, 2015).

Similar concerns emerged around rehoming research animals in the later stages of life. Ella, an NVS, explained that ‘given the trauma of the process of rehoming […] I think there is definitely a theoretical case where it might be better for them to be euthanised than rehomed if it’s going to be very traumatic and last for a short period of time’. NVS Megan added that while ‘I would like to say [rehoming is] great for the animals because they have a long life and they get to, you know … but I don’t really know. I don’t know if they necessarily value having a long life’. In other words, animals may lack awareness of any future time that is lost by their death; only the quality, not quantity, of life may matter to them (Rollin, 2011). Humane killing was therefore sometimes viewed as better than rehoming for the animals themselves, if their future was imagined to involve suffering or if length of life was imagined to be irrelevant from the animal’s perspective.

Similar concerns about the extension of life causing unnecessary suffering emerged in veterinary clinical trials. For example, researcher Charles described a disagreement with his former Home Office inspector over a proposed clinical trial for a terminal illness treatment in companion animals. Charles explained that the inspector’s initial response to the project application was ‘like, you thought I’d asked to do, you know, the most cruel thing ever’. Reflecting on the inspector’s perspective, Charles observed that, ‘There are some vets out there that think, as soon as you have [this illness] in a patient you should probably put them to sleep’. In other words, the inspector felt that euthanasia would have been better for any pet than participating in Charles’s clinical trial.

Several participants observed that this preference for euthanasia was most often expressed by veterinary anaesthetists, perhaps because, as one anaesthetist noted, their work directly involves assessing and minimising suffering (AP field notes, 18 January 2019). For example, another anaesthetist described being ‘horrified’ by a particular clinical study she’d witnessed, on account of ‘the amount of suffering these animals went through’ (interview with AP). However, the anaesthetist was broadly supportive of laboratory animal research, and was specifically concerned about clinical research due to a perceived lack of oversight of some experimental treatments (Palmer et al., 2020, 2021) and informed consent of owners. One vet even speculated that it may never be ethical to conduct experimental research with pets where the outcomes are truly unknown or expected to cause suffering (AP field notes, 14 January 2019). The fact that clinical research involved *pets* was therefore viewed as ethically significant. Furthermore, these participants suggested that the legal, social, and professional norms in place in the laboratory make it a more ethically appropriate place to use animals for science than the clinic. Charles’s experience suggests that these principles carry through into the implementation of A(SP)A, since his project would likely have faced no controversy if he had proposed to conduct it with laboratory animals, but he faced difficulty getting it approved for pets.

These concerns are closely related to a broader set of debates within the veterinary profession about ‘heroic treatments’, i.e. treatments perceived to cause unnecessary suffering compared with standard treatment or euthanasia (Clutton et al., 2017; Grimm et al., 2018; Sandøe et al., 2016). For example, veterinarian Gillian observed that prosthetic limbs for cats and dogs tend to cause pain, from multiple fitting sessions and discomfort from foreign material introduced into the animal’s body. Meanwhile, cats and dogs are known to do well with only three legs, so amputation is usually simpler, cheaper, and less painful (AP field notes, 26 October 2018). Some vets argue that there is a growing trend within the profession towards heroic treatments, brought about by an increasing focus on profit, career development, and avoiding litigation (Sandøe et al., 2016), and a growing tendency for animal owners to request life-extending treatments no matter the cost (Clutton et al., 2017; Grimm et al., 2018; Rollin, 2011). Two older vets expressed the idea that there is a generational difference in the veterinary profession, such that younger vets tend to less frequently endorse euthanasia (AP field notes, 29 November 2018; 18 January 2019). Veterinary clinical research was sometimes framed as part of this broader trend towards extending the lives of companion animals, even when doing so causes suffering. This trend may be demonstrated in the growth in veterinary palliative care services (Dyson, 2015).

### Extending life

But for others, extending life is often preferable to humane killing, one justification being that death can harm animals’ welfare by preventing them from enjoying positive experiences they would have had otherwise. Thus, you could argue that while some deaths are in the animal’s interest – if their remaining life is expected to contain far more negative than positive experiences – death is not necessarily neutral for the animal, and can sometimes be considered a harm (Yeates, 2010). If euthanasia is justified when an animal’s life is not worth living, you could equally argue that animals should not be killed if they have a life worth living (Yeates & Main, 2009). As animal experimentation is framed to the public as only necessary due to the lack of alternatives, the same is arguably true of killing (Franco & Olsson, 2016), i.e. you could argue that the ‘default’ should be to extend life rather than humanely kill.

A second potential reason for preferring the extension of life over euthanasia is the idea of an animal’s life as valuable in and of itself. Rehoming is sometimes justified as imparting (at least some) laboratory animals an intrinsic value (Thierman, 2010). A common idea is that laboratory animals have ‘done their bit’ in aiding human health advances, meaning that they should be provided with a prolonged and high-quality life outside of the laboratory (Fleury, 2017). As Chris, who was previously responsible for developing a laboratory dog rehoming scheme, explained, rehoming ‘reflects a culture of care and that the animals are seen as having their own intrinsic value’ (interview, 30 August 2018). Given their disproportionate representation as rehoming candidates (Skidmore & Roe, 2020), laboratory dogs, as Chris speaks about, and other traditional companion species might be most commonly ascribed intrinsic value.

The idea of animal life as intrinsically valuable can also be used to justify the notion that allowing suffering is an inevitable element of care. Drawing on the veterinary palliative care practices of the Skanda Vale spiritual community in Wales, Hurn and Badman‐King (2019) argue that ‘[t]he mere condition of being alive, of leading a life to the end of its natural course, is singularly important’ (p. 143). On these grounds, the authors argue that good care involves allowing animals to experience a ‘natural death’ and caring for them while this occurs. In this framing, ‘good care’ is incompatible with euthanasia, but may involve considerable suffering: palliative care might be ‘beautiful’ but ‘it can also be incredibly violent, even in cases involving minimal pain and physical suffering’ (p. 152). Thus, both euthanasia and extending life can involve juxtaposing care with violence. At play in shaping views on this subject are not only norms – embedded, for example, in animal research law and veterinary professional standards – but also people’s roles and personal interactions with animals, as demonstrated by the tendency for veterinary anaesthetists to be especially worried about suffering. Views on extending versus ending animal life are also shaped by people’s personal values (e.g., around the intrinsic value of animal life) and how they imagine an animal’s future.

## Conclusions

To conclude, we reflect on what lessons our case studies can offer for geographies of animal care, death, and categories.

### Care

Our research highlights that UK animal research law seeks to split off the ethical from the affective and practical elements of care. This splitting of care is founded on the idea that intimate bonds developed through daily care compromise decision-making about animal care and death, an assumption echoed in other contexts such as conservation (Palmer, 2020). This assumption could be challenged, for example, using arguments made in geographies of expertise, and emotion and affect (Pile, 2010): that such bonds are not only important, but also potentially a valuable form of knowledge in themselves. Regardless, many of our participants supported this splitting of care. Furthermore, it is worth noting that while there is no such split in the governance of pets, this is not a product of bonds and intimacy being legally respected. Rather, it results from pets’ status as property and therefore under the control of their owner. Care of pets therefore does not offer a particularly useful alternative model.

A second implication for care highlighted in our research was to reiterate the importance of attending to care in practice, not just in regulations or standards (see, Greenhough & Roe, 2018; Singleton, 2015). As we demonstrated, care regularly deviates from formal standards; for example, both animal owners and scientists are sometimes brought in on euthanasia decisions in veterinary clinical research despite technically having no say. Such deviations may lead to better care, but not necessarily. Furthermore, care might technically fulfil legal requirements (e.g., that owners sign a consent form) while failing to deliver on the principles (e.g., that consent is truly free and informed). This suggests not only that what ‘good care’ looks like is context-dependent, and that establishing universal rules for good care is problematic (Harbers, 2015), but additionally highlights the importance of subtle spatial, social, and other elements of ‘workplace atmospheres’ that can contribute towards a ‘culture of care’ (Greenhough & Roe, 2018; Roe & Greenhough, 2021).

Finally, our work supports Puig de la Bellacasa’s (2017, p. 66) argument that care is speculative, and future- as well as present-oriented. In both rehoming and clinical trials, expectations of future suffering, and positive experiences, were important in assessments of whether extending the animal’s life was justified. Speculating about future suffering, and hence care, was easier in some circumstances than others, with the laboratory readily viewed as a site of more standardised care than the home. Such uncertainties led to practices aimed at making better guesses about the future (e.g., inspections of potential rehomers’ living circumstances). Uncertainties around future suffering post-rehoming also underpin the European Directive’s (2010/63/EU) construction of death as the ‘default’ if future suffering cannot be reasonably ruled out.

### Death

Our case studies also speak to the important, but under-studied, subject of animal death (Mazhary, 2021) by highlighting principles embedded in the laboratory and veterinary clinic. Our research highlights the need to complicate the simplistic narrative that pets are killed more appropriately than laboratory animals, since pets are treated as ‘patients’ and only killed when they are suffering, while laboratory animals are killed ‘pragmatically’ and for the greater good of science. Animal owners, and vets too, may wish to end of extend pets’ lives for reasons other than the patient’s best interests (e.g., finances, career advancement, spending more time with a companion). Meanwhile, research animals are typically killed for science, but in some cases – such as rehoming and clinical research – there is an imperative to put the animal first. Care in the laboratory is also often perceived as more standardised and certain, and some laboratory staff and vets take pride in knowing that in the laboratory animal suffering is never extended purely for emotional reasons. Practices of killing in both laboratory and clinic are therefore not governed by a single ethical principle or norm, but rather are variable and dependent on spatial, social, and professional factors.

### Categories

Our research therefore suggests that pets do not necessarily have better lives or deaths than research animals: that depends on the specific context, and on personal ethical judgements such as whether suffering is worse than death. This means that while pets are typically ascribed higher ranks on ‘sociozoologic’ and ‘zoometric’ scales than research animals (Arluke & Sanders, 1996; Braverman, 2017), this does not necessarily mean they receive better care and welfare.

We have additionally highlighted the changing nature and flexibility of animal classifications. Professional, social, and legal changes mean that the animal welfare implications of categorisation shift over time; norms around ending and extending life for pets are on the move, according to some of our research participants. Categories are also highly flexible, particularly in liminal contexts. For example, certain elements of a previous classification may readily ‘stick’ where animals shift between categories, such as when animal owners are made to feel included in decisions about care and euthanasia in clinical trials even when their consent is no longer legally required. This might occur via deployment of classic ‘skills of diplomacy’ used in the veterinary clinic, or researchers and regulators together ensuring that the only clinical research that takes place involves animals for whom euthanasia is highly unlikely or an inevitability, thus minimising conflicts between vets and animal owners. Furthermore, our research highlights how socio-legal categories intersect in important ways with species categories; for example, it is particularly easy to treat research dogs as pets (Giraud & Hollin, 2016) and hence to rehome them and view them as intrinsically valuable.

These complexities highlight the need for further research investigating the details of how and why animal classifications and hierarchies emerge, the animal welfare implications of classification, and the limitations to thinking categorically given the complexities of real-world human-animal interactions. As we have shown, borders and transition zones between animal categories are particularly valuable places for investigating these matters.

## Data Availability

By agreement with the Wellcome Trust and research participants, anonymised interview transcripts will be deposited in the UK Data Archive based at the University of Essex (https://www.data-archive.ac.uk) after a period of 10 years from the completion of the Animal Research Nexus Project in 2022, except in cases where participants have explicitly opted out of this arrangement.

## Note

1. Now in the School of Biological Sciences and School of Social Sciences, University of Auckland, Auckland, New Zealand.
